# Study on the Mechanism of Formononetin Against Hepatocellular Carcinoma: Regulating Metabolic Pathways of Ferroptosis and Cell Cycle

**DOI:** 10.3390/ijms26062578

**Published:** 2025-03-13

**Authors:** Ning Bao, Zichao Chen, Baohong Li, Haolin Yang, Xiao Li, Zhen Zhang

**Affiliations:** 1 Innovation Research Institute of Traditional Chinese Medicine, Shandong University of Traditional Chinese Medicine, Jinan 250355, China; bn990918@163.com (N.B.); libaohong1019@163.com (B.L.); hlin18006399972@163.com (H.Y.); 2 Experimental Center, Shandong University of Traditional Chinese Medicine, Jinan 250355, China; chenzichao11@126.com

**Keywords:** formononetin, hepatocellular carcinoma, ferroptosis, cell cycle arrest, metabolomics, network pharmacology

## Abstract

Formononetin (FM), an isoflavone with a range of anti-cancer activities, has not been fully elucidated regarding its anti-hepatocellular carcinoma (HCC) mechanisms. Therefore, this study aims to explore the underlying mechanisms of FM using a comprehensive pharmacology model based on computational technologies and omics technology. A network pharmacology approach was applied to detect the components and targets. A mathematical formula was used to evaluate the network contribution index (CI). Bioinformatics analysis was used to analyze clinical data related to HCC targets corresponding to the core component, and molecular docking simulations were conducted to assess binding activity. The results showed that FM induces oxidative DNA damage through ROS generation and triggers G2/M phase cell cycle arrest via the Chk1/Cdc25C/CDK1/CCNB1 signaling pathway. Subsequently, UPLC-MS/MS was applied for the analysis of differential metabolites and the exploration of distinct metabolic pathways. FM limited the synthesis of glutathione, promoted lipid peroxidation, and facilitated the generation of divalent iron. Finally, a colony formation assay, Western blot, and molecular dynamics simulation methods were executed to further validate the metabolomic results. FM exhibited a strong binding affinity for glutathione peroxidase 4 (GPX4). In addition, FM induces ferroptosis by inhibiting the p53/xCT/GPX4 signaling pathway. In vivo, FM could inhibit tumor growth. Conclusions: FM could induce DNA damage leading to cell cycle arrest and may also induce ferroptosis by regulating glutathione metabolism, thereby intervening in the occurrence and development of HCC, making it a promising candidate for HCC treatment.

## 1. Introduction

Liver cancer is a worldwide health challenge and hepatocellular carcinoma (HCC) is the main type [1]. Over the past two decades, there has been a substantial increase in the occurrence of HCC, making it the fifth most prevalent malignancy reported by the World Health Organization. Therefore, effective drug research and the discovery of mechanisms of action are particularly urgent.

Developing more effective and safer natural products has gradually become a mainstream strategy for cancer treatment. Natural compounds possess broad biological activities and can safely and effectively treat tumors. They also exhibit stronger biocompatibility and lower cytotoxicity compared to chemically synthesized drugs, making these pharmacological advantages particularly crucial in cancer treatment [2]. It is reported that about 75% of current anti-cancer drugs are plant-derived natural products, such as paclitaxel, vincristine, and vinblastine [3]. Formononetin (FM) is a phytoestrogen isoflavone widely found in dietary sources [4]. These phytoestrogens belong to secondary plant metabolites and are known in the food and pharmaceutical fields for their biological functions, including antioxidant, anti-inflammatory, and neuroprotective effects [5]. In traditional Chinese medicine, *Astragalus mongholicus* Bunge (Huangqi) is widely used to extract FM and is clinically applied in the treatment of various tumors, including lung cancer, liver cancer, and breast cancer [6]. It has been documented that populations who consume soy products with a higher concentration of plant estrogens demonstrate a reduced prevalence of breast cancer [7].

In recent years, FM has gradually become a research hotspot due to its anti-cancer potential, with most studies indicating that the half maximal inhibitory concentration (IC50) of FM in exerting anti-cancer effects ranges from 10 to 300 μM, showing inhibitory effects on various cancer cells [8]. FM can exert its anti-tumor effects by inhibiting cell cycle progression, inducing apoptosis, and suppressing tumor angiogenesis. In vivo, FM can also exhibit significant tumor growth inhibition and can inhibit tumor invasion and angiogenesis, typically administered via intraperitoneal injection at doses of 10–60 mg/kg [5]. Studies have shown that treatment with 20–40 mg/kg of FM effectively inhibits the growth of human multiple myeloma subcutaneous xenografts in nude mice [9]. Additionally, FM may exert its anti-tumor effects by inducing reactive oxygen species production and disrupting the cellular antioxidant system. After using antioxidants such as glutathione, the inhibitory effect of FM on human multiple myeloma cells is diminished [9]. Furthermore, FM can disrupt the balance of glutathione (GSH) and oxidized glutathione (GSSG). The imbalance in the GSSG/GSH ratio directly limits its ability to remove reactive oxygen species (ROS) and results in an increase in ROS production, leading to the occurrence of oxidative stress [9].

Currently, due to the rapid development of computer technology, various omics and systems pharmacology methods are being employed to detect the mechanisms of action of biomolecules [10]. Network pharmacology has been recognized as a powerful tool for exploring the interactions between active molecules and disease targets, while theoretical methods based on molecular simulation have become one of the primary means to study the functions of biological macromolecules such as proteins [11,12]. The rise in metabolomics technology has enabled researchers to more accurately identify and elucidate the biological effects brought about by drug treatments [13,14]. Bioinformatics analysis, utilizing genomic data for the analysis and clinical interpretation of tumors, has proven instrumental in identifying and validating novel drug targets [15,16]. Combining network pharmacology with metabolomics helps to explore the relationship between metabolic products in liver cancer and the action targets of FM, enhancing our understanding of the complex mechanisms of action between FM and HCC, thereby making it a promising candidate for HCC treatment.

In this study, we demonstrated the anti-HCC activity of FM. By constructing a topology network based on bioinformatics, network pharmacology, metabolomics technology, molecular dynamics (MD) simulation, and molecular biology methods, we found that FM could cause oxidative DNA damage by promoting the generation of reactive oxygen species and arresting the cell cycle at the G2/M phase through the Chk1/Cdc25C/CDK1/CCNB1 signaling pathway. FM induces ferroptosis by inhibiting the p53/xCT/GPX4 signaling pathway. These results suggest that FM may serve as a potential candidate for the treatment of HCC.

## 2. Results

### 2.1. Network Pharmacology Prediction and Core Component Identification

**Figure 1:**
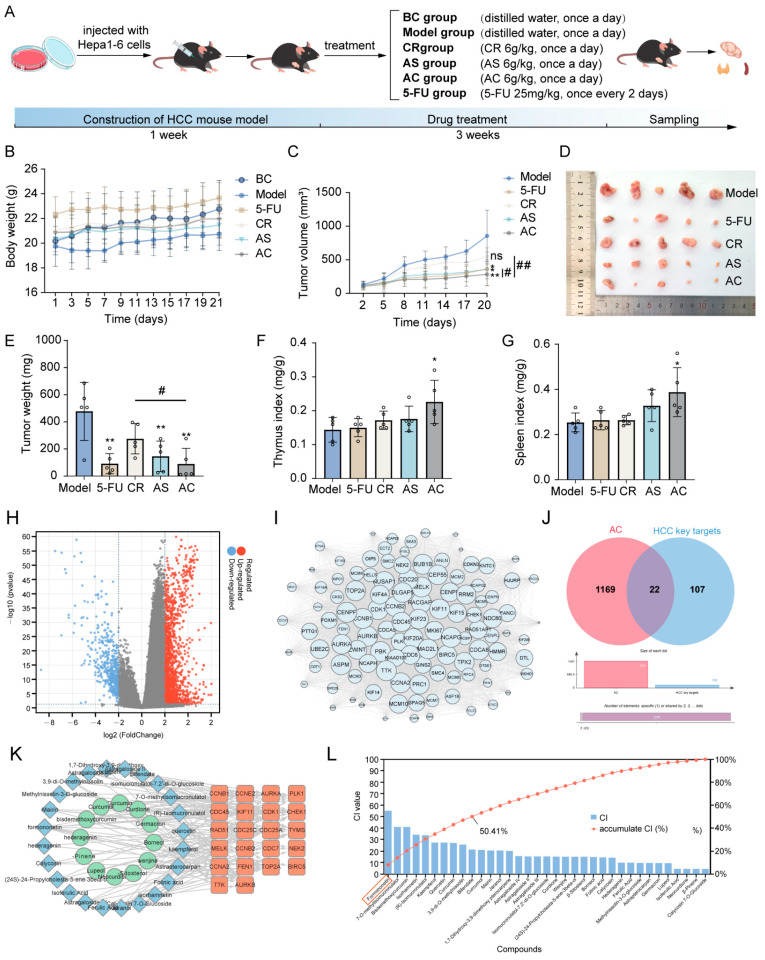
Inhibition of tumor growth in HCC model mice. (**A**) The treatment of each group. (**B**) The changes in body weight of mice in each group. (**C**) The tumor growth status of mice in each group. (**D**) The morphology of subcutaneous tumors. (**E**) The tumor weight of mice in each group. (**F**) The thymus index of mice in each group. (**G**) The spleen index of mice in each group; *n* = 5. (**H**) Significantly differentially expressed genes are shown in a volcano plot with *p* < 0.05 and |log2FC| > 2; (**I**) 129 key targets of the PPI network. (**J**) The Venn diagram of AC targets and HCC key targets. (**K**) The component–target network of AC. (**L**) The CI value of each component. Data are expressed as mean ± SD. * *p* < 0.05 and ** *p* < 0.01; # *p* < 0.05 and ## *p* < 0.01, ns means not significantly.

During the treatment period, there was no significant difference in the growth trend of the body weight across all groups (Figure 1A,B). The growth curve and tumor mass of the tumors in each group are shown in Figure 1C–E. The results showed that compared with the model group, *Astragalus mongholicus* Bunge (AS), *Curcuma aromatica* Salisb. (CR), *Astragalus mongholicus* Bunge-*Curcuma aromatica* Salisb. (AC), and 5-Fluorouracil (5-FU) all had inhibitory effects on tumor growth in tumor-bearing mice. Additionally, the AC group exhibited more significant anti-tumor effects compared to the AS and CR groups (*p* < 0.05 and *p* < 0.01). Figure 1F, G shows the changes in spleen index and thymus index after AC and 5-FU treatment. Compared with the model group, the spleen index and thymus index of the AC group were significantly increased (*p* < 0.05), and there was no significant difference between other groups. These results indicated that AC could inhibit the growth of tumors in tumor-bearing mice. Next, we aimed to identify the active components through network pharmacology methods. A total of 1847 differentially expressed genes (DEGs) associated with HCC were identified. These DEGs were visualized in a volcano plot, with red dots representing upregulated genes and blue dots representing downregulated genes (Figure 1H). Subsequently, 129 key targets of the (protein-protein interaction) PPI network were identified through the MCODE of Cytoscape software 3.9.1 (Figure 1I, Table S1). A total of 36 active compounds and 175 targets were screened, as well as 22 anti-HCC targets with HCC (Figure 1J,K, Tables S2 and S3). Then, a mathematical formula based on network topology parameters was performed to calculate the CI of each compound. As shown in Figure 1L, the top ten compounds with an accumulated CI value of 50.41% are as follows: Formononetin, 7-O-methylisomucronulatol, bisdemethoxycurcumin, isorhamnetin, (R)-isomucronulatol, kaempferol, quercetin, curcumin, 3,9-di-O-methylnissolin, and bifendate, which can contribute the greatest effect of AC on HCC. FM, as one of the isoflavones in AS, has been reported to possess various anti-cancer effects [5].

### 2.2. Bioinformatics Analysis and Molecular Docking Analysis

**Figure 2:**
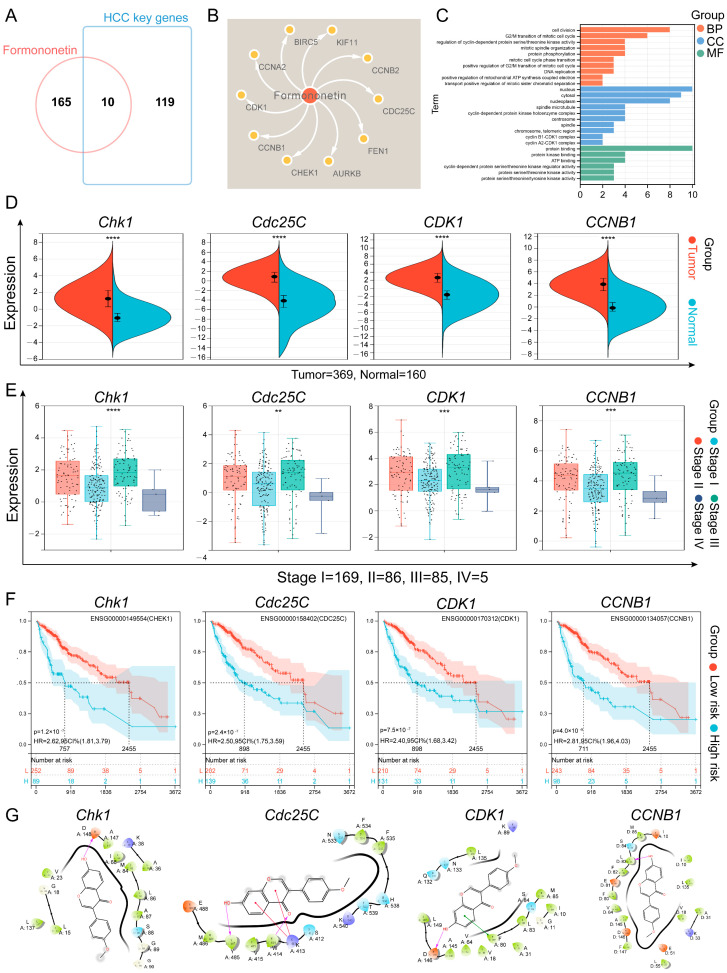
Bioinformatics analysis and molecular docking analysis. (**A**) The Venn diagram of FM targets and HCC key genes. (**B**) The component–target network of FM. (**C**) The GO results of core targets. (**D**) Different expression levels of core targets between normal and HCC tissues. Red represents tumor tissue and blue shows normal tissues, **** *p* < 0.0001; (**E**) Different expression levels of core targets at different stages of HCC, ** *p* < 0.01, *** *p* < 0.001 and **** *p* < 0.0001; (**F**) Survival analysis curves for the core targets in HCC patients. (**G**) Two-dimensional interaction diagrams of molecule docking.

Next, we collected the targets of FM’s anti-HCC effects (Figure 2A,B) and conducted Gene Ontology (GO) pathway enrichment analysis. The results showed that the core targets were involved in biological processes such as the cell division and G_2_/M transition of the mitotic cell cycle, etc. (Figure 2C), which suggests that FM may exert its anti-HCC effects by regulating the processes of the cell cycle. To clarify the role of core targets in the progression of HCC, we analyzed the clinical information of the core targets using bioinformatics methods. According to an analysis of the GEPIA database, the expressions of these core targets between HCC and normal tissues show significant differences (Figure 2D and Figure S1A). The analysis of HCC pathological staging showed that there were significant differences between the expression levels of these FM-regulated targets in normal tissues and tumor tissues and the pathological staging (Figure 2E and Figure S1B). Furthermore, a survival study revealed that the expression of core targets is significantly correlated with the survival time of HCC (Figure 2F and Figure S1C). Molecular docking is one of the powerful tools for predicting the binding affinity of active components with potential targets. The candidate target proteins Chk1, Cdc25C, CDK1, CCNB1, CCNA2, KIF11, AURKB, FEN1, and BIRC5 (PDB ID: 5oq7 2.10 Å, 3op3 2.63 Å, 4yc6 2.6 Å, 5lqf 2.06 Å, 7mkx 3.08 Å, 4a51 2.75 Å, 4af3 2.75 Å, 5fv7 2.84 Å, 2rax 3.3 Å) and CCNB2 (O95067) conducted molecular docking with FM, and the results show that these targets had a strong favorable bond (Figure 2G and Figure S1D). The docking scores are as follows: Chk1: −7.015, Cdc25C: −5.317, CDK1: −5.802, CCNB1: −7.888, CCNB2: −5.012, CCNA2: −5.858, KIF11: −6.091, AURKB: −7.129, FEN1: −4.675, and BIRC5: −3.487. Interaction information between the targets and FM is presented in Table 1. Collectively, these results demonstrated that FM may suppress HCC by disrupting the cell cycle by acting on multiple cell cycle proteins.

### 2.3. FM Inhibits the Proliferation of HepG2 Cells

**Figure 3:**
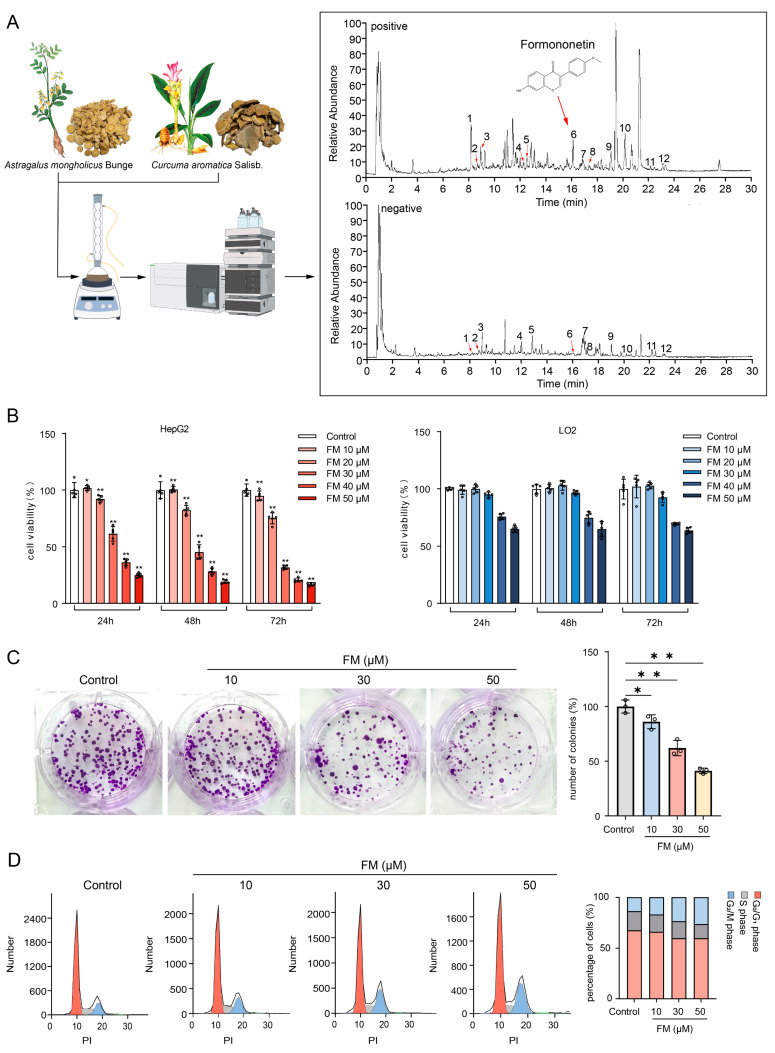
FM inhibits the proliferation of HepG2 cells. (**A**) Identification of FM (1: Ferulic acid, 2: Isoferulic acid, 3: Calycosin-7-O-beta-D-glucoside, 4: Methylnissolin-3-O-glucoside, 5: Calycosin, 6: Formononetin, 7: Astragaloside IV, 8: Astragaloside III, 9: Astragaloside II, 10: Curdione, 11: Bisdemethoxycurcumin, and 12: Curcumol); (**B**) MTT assay, *n* = 5; (**C**) colony formation assay, *n* = 3; (**D**) FM induces G_2_/M arrest, *n* = 5. Data are expressed as mean ± SD. * *p* < 0.05 and ** *p* < 0.01 vs. control group.

The total Ion Chromatogram of AC water decoction is shown in Figure 3A, and FM was identified from it. The viability of HepG2 and LO2 cells treated with FM was evaluated using the MTT assay. In Figure 3B, the inhibition of FM on the growth of HepG2 cells significantly increased in a dose- and time-dependent manner, with half-maximal inhibitory concentrations (IC_50_) at 24 h, 48 h, and 72 h being 36.57 μM, 28.68 μM, and 25.93 μM. Notably, FM did not show a significant impact on the growth of LO2 within 10–30 μM. Then, the colony formation assay demonstrated that FM significantly inhibited the clonogenesis of HepG2 cells (Figure 3C). These findings collectively suggest that FM possesses the ability to suppress HepG2 cell proliferation. Bioinformatic analysis suggests that the effect of FM may be related to the cell cycle pathway. Therefore, flow cytometry was employed to study the cell cycle distribution. In Figure 3D, FM treatment altered the cell cycle distribution of HepG2 cells, and as the FM concentration increased, the proportion of cells in the G_2_/M phase increased, indicating that FM can induce the cell cycle arrest of HepG2 cells in the G_2_/M phase, which is consistent with the results of the GO analysis.

### 2.4. FM Induces DNA Damage by Accumulating ROS

**Figure 4:**
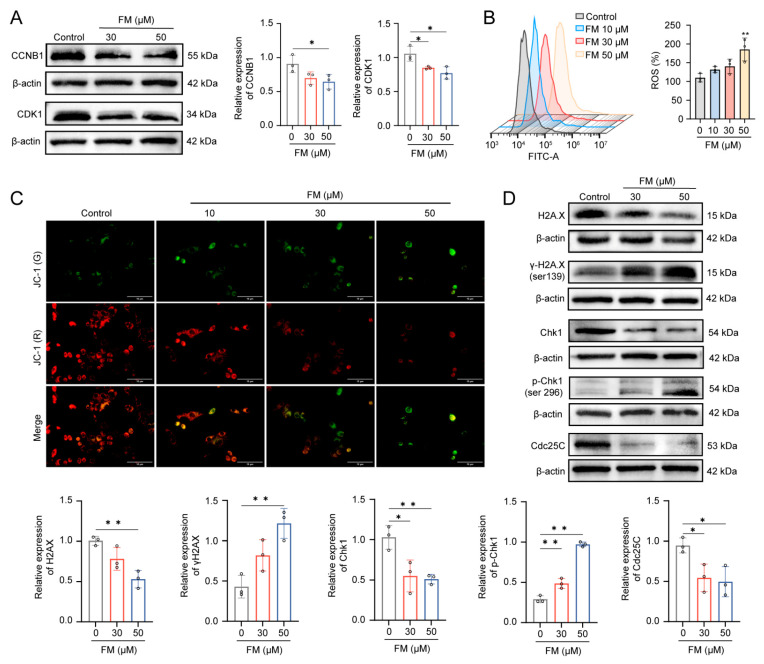
FM induces DNA damage by accumulating ROS. (**A**) FM decreases the expression levels of CCNB1 and CDK1. (**B**) ROS levels of HepG2. (**C**) MMP of HepG2 cells stained by JC-1 (Scale bar: 10 μm); (* *p* < 0.05, **p < 0.01). (**D**) Western blotting analysis of H2A.X, γ-H2A.X, Chk1, p-Chk1, and Cdc25C after treatment of FM. *n* = 3. Data are expressed as mean ± SD. * *p* < 0.05 and ** *p* < 0.01 vs. control group.

To verify the results of cell cycle detection, the WB experiment was used to detect the expression of the G_2_/M phase transition proteins. The reduced expression of CDK1 and CCNB1 indicates the arrest of the G_2_/M phase (Figure 4A). It has been reported that the accumulation of ROS is one of the factors causing DNA damage. Fluorescent microscopy and flow cytometry results revealed that FM could induce the accumulation of ROS and a decrease in MMP in HepG2 cells in a dose-dependent manner (Figure 4B,C). Subsequently, the occurrence of DNA damage was assessed by the expression of the DNA damage marker H2A.X and the activation of the checkpoint proteins Chk1 and CDC25C. As shown in Figure 4D, the results revealed a significant down-expression of H2A.X and an up-expression of γ-H2A.X, which indicated acute oxidative stress. Then, Chk1 and Cdc25C were activated by respective phosphorylation modifications. Collectively, these findings indicate that DNA damage activates the Chk1/Cdc25C/CDK1/CCNB1 pathway, which in turn can lead to cell cycle arrest.

### 2.5. FM Regulates Cellular Metabolism

**Figure 5:**
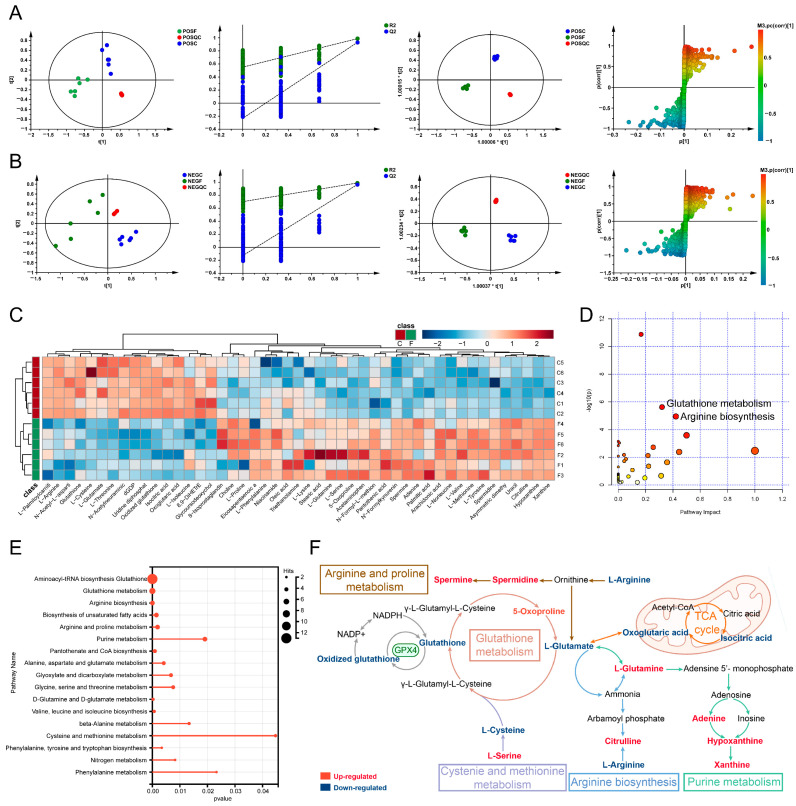
FM regulates cellular metabolism. (**A**,**B**) PCA score plots, PLS-DA model validation diagram, OPLS-DA score plots, and S-plot of OPLS-DA. POS: positive modes; NEG: negative modes; F: FM group; C: control group; QC: quality control. *n* = 3. (**C**) Hierarchical clustering analysis heatmap; (**D**–**F**) metabolic pathway enrichment analysis, the colors in figure D (varying from yellow to red) means the metabolites are in the data with different levels of significance.

To further explore the mechanism of FM anti-HCC, UPLC-MS/MS was employed to assess the metabolomics profiles of HepG2 cells. The base peak chromatograms were analyzed in both positive and negative modes (Figure S2). According to PCA analysis, the FM group was separated from the control group, and clustering QC confirmed the stability of the instrument. The permutation test showed that the PLS-DA model validation was valid. The differential metabolites (R^2^X (0.597/0.62), R^2^Y (0.988/0.987), and Q^2^ (0.964/0.93)) were found using the PLS-DA model. S-plots showed the effects of different variables (Figure 5A,B). Consequently, 47 differential metabolites were identified (Figure S3; Table 2). A total of 31 metabolites (e.g., L-Glutamate, L-Serine, 5-Oxoprolin, arachidonic acid, and oleic acid) significantly increased, whereas 16 metabolites (e.g., oxidized glutathione, glutathione, and L-cysteine) significantly decreased in the FM group. A hierarchical clustering analysis heatmap illustrated distinct clustering between the FM and control groups (Figure 5C). The metabolic pathway enrichment revealed that after FM treatment, the glutathione metabolism, cysteine and methionine metabolism, arginine biosynthesis, and so on were regulated (Figure 5D–F).

### 2.6. FM Induces Ferroptosis in HepG2 Cells

**Figure 6:**
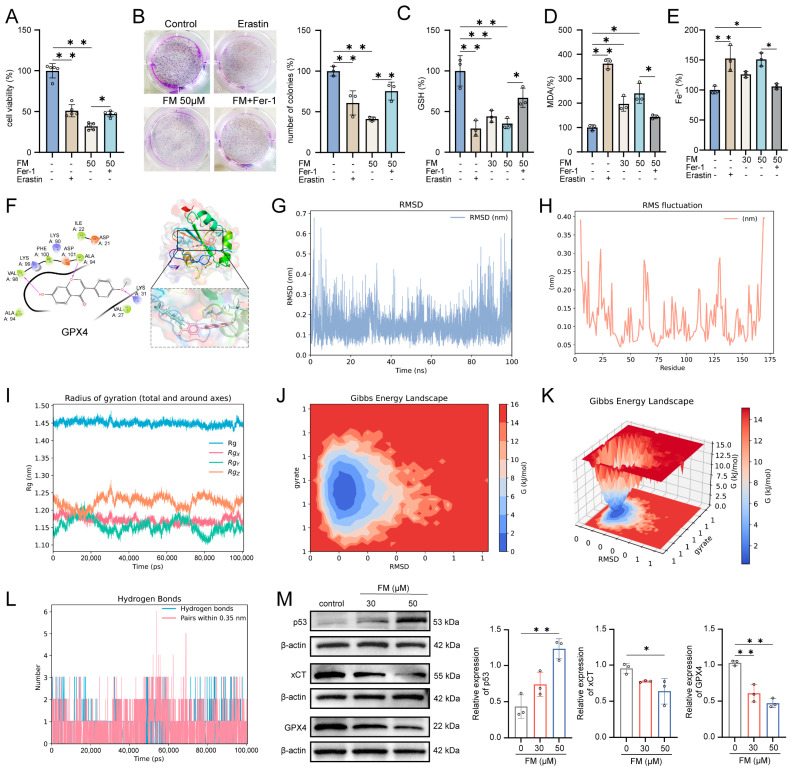
FM induces ferroptosis in HepG2 cells. (**A**) Fer-1 rescues the inhibitory effect of FM HepG2 cell viability; (**B**) Fer-1 rescues the inhibitory effect of FM HepG2 cell clonogenesis; (**C**) GSH, (**D**) MDA, and (**E**) Fe^2+^ levels in different groups; (**F**) 2D and 3D diagrams of molecular docking; (**G**) RMSD, (**H**) RMSF, and (**I**) RG diagrams; (**J**,**K**) Gibbs free energy profiles based on RMSD and gyration radius calculations; (**L**) hydrogen bond number curve; (**M**) p53, xCT, and GPX4 expression levels. *n* = 3. Data are expressed as mean ± SD. * *p* < 0.05 and ** *p* < 0.01 vs. control group.

Previous studies have demonstrated that FM promotes ROS accumulation and regulates glutathione metabolism. ROS is generated through the Fenton reaction between ferrous iron ions and hydrogen peroxide, leading to lipid peroxidation and ferroptosis [17], which indicates that FM may play a role in inducing ferroptosis. We assessed whether the inhibitory effect of FM on HepG2 cells involves ferroptosis by employing the ferroptosis inhibitor (ferrostatin-1, Fer-1) and the inducer erastin. Compared with the control group, treatment with Fer-1 (0–20 μM) had no significant change in cell viability, while treatment with erastin (0–20 μM) reduced the viability of HepG2 cells (Figure S4). In Figure 6A, both erastin (20 μM) and FM (50 μM) reduced HepG2 cell proliferation, while Fer-1 (10 μM) rescued proliferation. The colony formation assay confirmed that erastin and FM significantly inhibited HepG2 cell clonogenesis, which was rescued by Fer-1 (Figure 6B). GSH, MDA, and Fe^2+^ maintained oxidation–reduction balance and the levels changed during ferroptosis. GSH levels decreased with erastin and FM but were rescued by Fer-1 (Figure 6C). MDA and Fe^2+^ levels increased with erastin and FM but decreased with Fer-1 (Figure 6D,E). GPX4 plays a vital role in protecting against lipid peroxidation products, both of which are crucial in the ferroptosis process. To clarify the regulatory mechanism of FM on ferroptosis, molecular docking based on Maestro software 12.8 was first employed to investigate the potential binding activity of FM-GPX4, and the result showed strong binding, and the docking score was −4.454 (Figure 6F). To further explore how FM interacts with GPX4 in an aqueous environment under physiological conditions, a 100 ns MD simulation was run. The generated files were analyzed and visualized using DuIvyTools v0.5.0 software. RMSD provided the average distance between FM and GPX4 after the simulation, as well as precise data between the equilibrium structures after the simulation, which was used to evaluate the stability of the binding between the small molecule and the protein (Figure 6G). The structures of the GPX4-FM complex at the initial (0 ns) and final (100 ns) stages of the simulation were extracted and visualized using Pymol software 2.4.0 (Figure S5). The results showed that FM did not undergo significant displacement relative to the protein throughout the simulation, nor did it detach from GPX4 or show significant changes in the binding cavity, maintaining certain conformational changes within the binding cavity. RMSF was used in this study to analyze the flexibility and degree of motion of protein amino acid residues throughout the simulation. As shown in Figure 6H, the overall RMSF of the protein remained at a low level, indicating minimal protein fluctuation during the simulation, maintaining stable vibrations in the solution environment. The radius of gyration (RG) graph, combined with the previous analysis, indicates that the protein remained relatively stable throughout the 100 ns MD simulation, ensuring reliable sampling (Figure 6I). After eliminating the simulation trajectory, the binding free energy analysis was performed on the trajectory of the FM-GPX4 complex during the simulation. The Gibbs free energy landscape based on RMSD and the radius of gyration calculations was visualized (Figure 6J,K), and the energy terms were decomposed. The results showed that FM and GPX4 mainly interacted through van der Waals energy terms (−24.51 kcal/mol) (Figure S6, Table S4), with a low binding free energy between them, further explaining their binding stability from an energy perspective (Figure 6L). Western blotting results showed that the expression of p53, xCT, and GPX4 changed significantly (Figure 6M). These results collectively support the notion that FM induces ferroptosis in HepG2 cells through the p53/xCT/GPX4 pathway.

### 2.7. FM Inhibits Tumor Growth In Vivo

**Figure 7:**
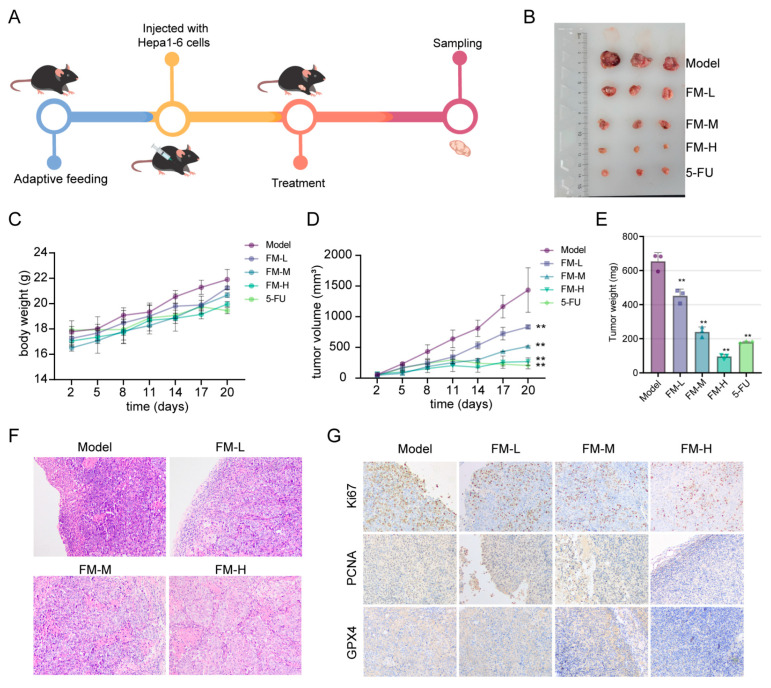
Inhibition of FM on tumor growth in HCC model mice. (**A**) Experimental procedure. (**B**) The morphology of subcutaneous tumors. (**C**) The changes in body weight of mice in each group. (**D**) The tumor growth status of mice in each group. (**E**) The tumor weight of mice in each group. (**F**) HE staining of tumor tissues. (**G**) IHC analysis of tumor samples. *n* = 3. Data are expressed as mean ± SD, ** *p* < 0.01 vs. control group.

The effects of FM on inhibiting tumor growth were validated through in vivo experiments. Hepa1-6 cells were injected into C57BL/6 mice to establish a subcutaneous xenograft tumor model. Once the tumor volume was measurable, FM and 5-FU were administered via intraperitoneal injection (Figure 7A,B). Figure 7C shows the changes in the body weight of the mice during the experiment. As shown in Figure 7D,E, during the treatment period, both 5-FU and FM significantly inhibited the growth of subcutaneous xenograft tumors, demonstrating a concentration-dependent effect. The results of HE staining also demonstrate the inhibitory effect of FM on HCC (Figure 7F). IHC results indicated that FM could downregulate the expression of proliferation markers Ki67 and PCNA, as well as the ferroptosis marker GPX4 (Figure 7G). These results collectively suggest that FM can inhibit tumor growth and induce ferroptosis, thereby exerting anti-HCC effects.

## 3. Discussion

Previous studies have indicated that the occurrence and development of HCC are accompanied by the interplay of multiple signaling pathways. Cell cycle regulation is closely linked to the uncontrolled cell proliferation of tumors. The key regulators of cell cycle processes are cyclins and cyclin-dependent kinases (CDKs) [18]. Ferroptosis, an iron-dependent form of cell death, is regulated by multiple metabolic pathways such as redox homeostasis and is associated with HCC [19]. Emerging evidence supports the idea that the dysregulated metabolic pathways play a role in the progression of HCC [20]. The solute carrier family 7 member 11 (SLC7A11) is a multi-pass transmembrane protein and a vital component of the cystine–glutamate antiporter, known as the xCT system. The majority of cancer cells depend on the xCT system to import cystine, which is then converted to cysteine. Subsequently, cysteine is utilized in the synthesis of GSH [21]. Therefore, inducing ferroptosis by targeting glutathione metabolism presents promising avenues for cancer treatment. Currently, small molecules that target these metabolic pathways have demonstrated the ability to induce ferroptosis. For example, ponicidin has been shown to induce ferroptosis by regulating the metabolites associated with glutathione metabolism in pancreatic cancer cells [22]. Polyunsaturated fatty acids in Quinoa have also been reported to reverse drug resistance in colorectal cancer by inducing ferroptosis [23].

FM, an isoflavonoid isolated from *Astragalus mongholicus* Bunge, has been confirmed to have anti-tumor effects. For instance, FM has been reported to inhibit the proliferation of various cancer cells, including colon, breast, and ovarian cancer cells. Its inhibitory effects on liver cancer cells have also been documented [8]. The inhibitory effects of FM on tumor growth are well documented; however, its mechanism for inducing cell death in HCC requires further investigation. To explore its molecular mechanisms, the TCGA sequencing data were used to identify DEGs in HCC and analyze the key genes associated with FM. GO enrichment analysis indicated that genes regulated by FM are mainly involved in the G_2_/M phase transition. The result was also confirmed by flow cytometry.

Abnormal ROS production is a common feature of cancer cells. The ROS accumulation led by mitochondrial membrane damage has been shown to promote cell cycle arrest [24]. Due to ROS influence, FM initiates a DNA damage response (confirmed by γ-H2A.X upregulation), which leads to the phosphorylation of Chk1 at Ser296. This phosphorylation directly inactivates Cdc25C phosphatase [25]. In the G_2_/M phase, the activation of CDK1 requires association with cyclin B. Subsequently, Cdc25C removes the inhibition of CDK1 imposed by related kinases. Thus, the activation of Chk1 and Cdc25C mediates G_2_/M checkpoint arrest, preventing CDK1 dephosphorylation and consequently leading to cell cycle arrest in the G_2_ phase [18]. Our findings demonstrate that FM disrupts this process through the dual mechanisms of sustaining Chk1 activation and suppressing Cdc25C activity, ultimately blocking CDK1 dephosphorylation. These data establish that FM specifically hijacks the Chk1/Cdc25C axis within the DNA damage checkpoint, creating a “molecular brake” on G2/M progression through the coordinated regulation of the CDK1 phosphorylation status and CCNB1 complex formation. 

In addition to inducing DNA damage, ROS accumulation can trigger various forms of cell death. Recent evidence underscores the necessity of mitochondrion-mediated ROS generation, DNA damage, and metabolic reprogramming in the induction of lipid peroxidation and ferroptosis [26]. Ferroptosis, characterized by metabolic disorders involving iron, lipids, amino acids, and ROS, has potential clinical therapeutic applications. Various natural products such as ursolic acid and β-elemene have been shown to achieve anti-tumor effects by inducing ferroptosis [27,28]. Enhancing anti-cancer efficacy by inducing ferroptosis is a viable approach for treating HCC. Ferroptosis involves multiple cellular metabolic processes, including the peroxidation of unsaturated fatty acid phospholipids, the formation of lipid peroxides, and glutathione synthesis. Therefore, to further investigate the correlation among FM, ferroptosis, and cellular metabolism, metabolomics was employed to analyze the differential metabolites in HepG2 cells treated with FM. In this study, two groups of cells showed a clear trend of separation in positive and negative ion modes. There was a notable identification of significantly distinct endogenous metabolites, primarily involved in glutathione metabolites and the biosynthesis of unsaturated fatty acids.

Cystine is a limiting amino acid in glutathione synthesis and the most abundant cellular antioxidant. Cystine synthesis is hindered due to reduced xCT activity, which subsequently leads to a decrease in GPX4 activity and finally induces ferroptosis and cell death [17]. Polyunsaturated fatty acids (PUFAs) play crucial roles in the various biological functions of tumor cells, and ferroptosis is caused by the iron-dependent accumulation of lipid peroxidation of PUFAs on the cell membrane [29]. A previous study also suggests that PUFAs like oleic acid and arachidonic acid can enhance the immunity of liver cancer rats to alleviate the symptoms of HCC [30]. The mechanisms of phospholipid hydroperoxides (PLOOHs), a type of lipid-derived ROS, and PUFAs (the precursor of PLOOHs) have been thoroughly examined in ferroptosis [31]. The subsequent results showed that, similar to the ferroptosis inducer erastin, FM could inhibit HepG2 cell proliferation and colony formation, while the inhibition was rescued by Fer-1. Ferroptosis-related indicators, such as GSH, Fe^2+^, and MDA expressions, significantly changed after FM treatment, with these changes rescued by Fer-1. Thus, we speculated that the inhibition of FM on HepG2 cells was also caused by inducing ferroptosis.

**Figure 8:**
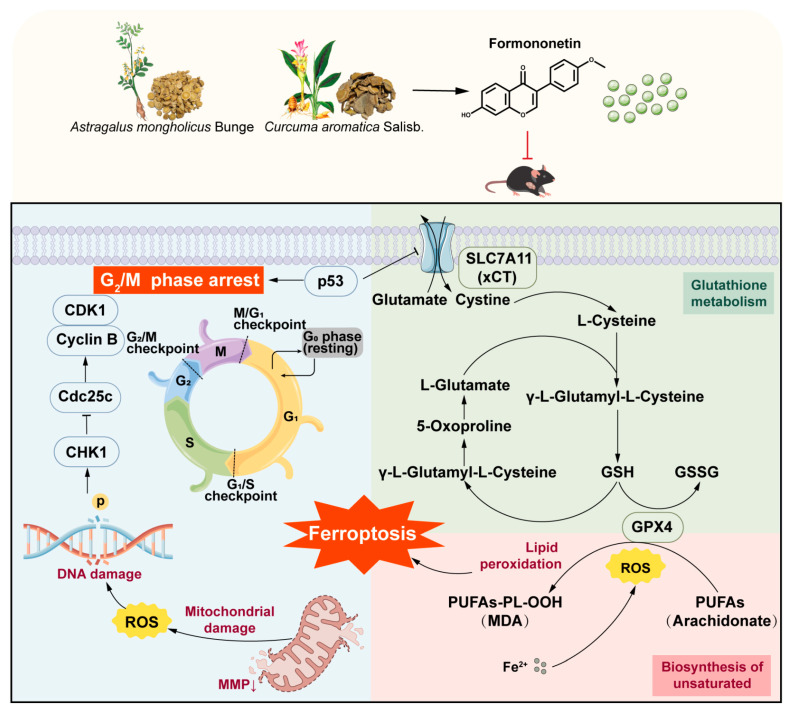
Molecular mechanisms of the anti-HCC effect of FM.

GPX4 is a confirmed key regulator of ferroptosis. To further verify the regulatory effect of FM on ferroptosis, molecular docking was used to investigate the binding activity of FM with GPX4. Then, the FM-GPX4 complex obtained from docking was used as the initial structure for a 100 ns all-atom MD simulation to verify its binding stability under simulated physiological conditions. This study found that FM did not dissociate from GPX4 or show significant binding pocket changes during the simulation, maintaining a stable complex with only minor conformational changes. Hydrogen bond analysis revealed consistent hydrogen bonding between FM and GPX4, further indicating binding stability. In summary, FM leads to the downregulation of xCT and GPX4, confirming that FM induces ferroptosis by inhibiting the p53/xCT/GPX4 pathway, which modulates oxidative stress and ferroptosis, targets tumor metabolic vulnerabilities, and serves as a broad-spectrum therapeutic target for cancers (Figure 8).

## 4. Materials and Methods

### 4.1. Network Pharmacology Analysis and Bioinformatics Analysis

#### 4.1.1. Data Sources and DEGs Analysis

The TCGA-LIHC data were obtained from the TCGA database. The differential expression analysis of HCC-related genes was conducted using the “limma” (3.50.3) and “edgeR” (3.30.3) software packages. The significance of differential gene expressions was assessed using the Wilcoxon rank-sum test, with a *p*-value < 0.05 and |log2FC| > 2. The volcano plots were generated using the “ggplot2” (3.3.6) software package.

#### 4.1.2. Identification of Key Gene Expression Modules

The PPI network was used to explore the interaction between DEGs by STRING database, and the interactions network was visualized utilizing Cytoscape 3.9.1 software. The MCODE plug-in of Cytoscape was employed to identify key gene expression modules with default settings.

#### 4.1.3. Collection of Active Components and Targets 

The active components came from the TCMSP database. Components were screened for subsequent analysis based on oral bioavailability (OB) ≥ 30% and drug-likeness (DL) ≥ 0.18. To identify the targets of the components, the TCMSP database, Swiss Target Prediction database, and SEA database were retrieved in this study. The PubChem database was used to catch the Isomeric SMILES document and the structural files of components. Second, they were input into these databases for prediction. The Venn diagram pack of R (4.1.3) language was used to capture the overlap of all targets and DEGs.

#### 4.1.4. Construction of Component–Target (C-T) Network

The screened components and corresponding anti-HCC targets were used to establish the C-T network using Cytoscape software (3.9.1). The network was used to detect the independently regulated and co-regulated targets.

#### 4.1.5. Core Component Identification

To identify the core component, a mathematical formula was used to evaluate the network contribution index (CI) of each component [32]

(1) $$\omega_{ei}=C_{edge}/T_{edge}$$

(2) $$A_{ij}=\omega_{ei}+|(C_{Ai}+C_{Bi})/(C_{Ai}-C_{Bi})|$$

(3) $$NE\left(i\right)=\sum_{ij}^{n}C_{i}\times \left[A_{ij}\times P_{j}\right]$$

(4) $$CI\left(i\right)=\left[NE\left(i\right)/\sum_{ij}^{n}NE\left(i\right)+C_{i}/\sum_{ij}^{n}C_{i}\right]\times 100$$

*i* represents component count, *j* represents target count, *ωei* represents the network node parameters, *Aij* is the index of affinity determined from the *ωei* value, *CAi* represents the degree of AS, *CBi* represents the degree of CR, *Ci* represents the degree of each component, *Pj* represents the degree of each target, and *NE* represents the network contribution of each component in the network; *Ci* represents the contribution of each component to the anti-HCC effect, and the parameters were obtained in the C-T network.

#### 4.1.6. Bioinformatics Analysis of Core Component

For the targets associated with FM and HCC, the DAVID database was applied for KEGG and GO enrichment analyses. Pathways with *p* values < 0.05 were retained for further consideration. The GEPIA database was applied to evaluate the different expression levels, staging, and overall survival analysis.

#### 4.1.7. Molecular Docking Studies

The structures of the core targets were obtained from the PDB and AlphaFold Protein Structure Database. Maestro version 12.8, as part of the Schrödinger suite 2021-2, was used for performing docking studies, with default settings. Lower docking scores represent stronger favorable bonds between proteins and ligands. Two-dimensional and three-dimensional interaction diagrams were generated using Maestro and Pymol.

#### 4.1.8. Molecular Dynamics Simulation

MD simulations were conducted using Gromacs 2023.2 under constant temperature and pressure conditions. The Amber14sb all-atom force field was employed for the protein, while the GAFF force field based on Amber was used for the small molecule [33,34,35]. The solvent system was water, and NaCl was used to neutralize the charge. After energy minimization of the system, a 0.5 ns NVT and NPT pre-equilibration simulation was performed. The parameters were set as follows: temperature of 298.15 K, pressure of 1 bar. A 100 ns MD simulation was then carried out, and the trajectory was saved every 5 ps. RMSD, RMSF, Hbonds, etc. of the protein were calculated and visualized using DuIvyTools v0.5.0 software (https://duivytools.readthedocs.io/en/latest/DIT_old.html, accessed on 1 March 2025). The detailed analytical methods are provided in the Supplementary Materials [36,37,38,39,40].

### 4.2. Experimental Validation

#### 4.2.1. Reagents

AS (production batch number: 2023021327) and CR (production batch number: 22040108) were purchased from Anhui Xiehecheng Pharmaceutical Co., Ltd. (Bozhou, China). FM (purity > 98%), erastin, and ferrostatin-1 (Fer-1) were purchased from Shandong Sparkjade Biotechnology Co., Ltd. (Jinan, China). 5-FU was purchased from the MedChemExpress Biotechnology Company (Monmouth Junction, NJ, USA) and used as the positive control.

Cyclin B1 (CCNB1), cyclin-dependent kinase 1 (CDK1), checkpoint kinase 1 (Chk1), Phospho-Chk1, cell division cycle 25C (Cdc25C), p53, Histone H2A.X, Phospho-Histone H2A.X (Ser139), glutathione peroxidase 4 (GPX4), xCT (solute carrier family 7 member 11), and β-actin Recombinant Rabbit mAb (db13323, db12527, db14895, db7475, db2109, db12023, db11980, db13191, db14187, db12720, and db13986) were purchased from Diagbio (Hangzhou, China); and secondary antibodies were purchased from Abclonal (Wuhan, China).

#### 4.2.2. Identification of Main Components

In total, 400 g of AS, 400 g of CR, and 600 g of AC herb pair (2:1) were soaked in 10 times the amount of water for 1 h, refluxed for 0.5 h, and extracted twice, and the volatile oils were collected. The combined liquid was rotary evaporated and concentrated to a concentration of 1 g·mL^−1^. UPLC/MS was used for drug quality control. Thermo Scientific™ U3000-Q Exactive Focus (Thermo, Waltham, MA, USA) was used with the Hypersil GOLD chromatographic column (100 × 2.1 mm, 1.9 μm) (Thermo, Waltham, MA, USA), with a column temperature of 40 °C. The flow rate was 0.3 mL/min and the injection volume was 5 μL. The mobile phases were 0.1% formic acid in water (A) and 0.1% acetonitrile (B). The gradient elution conditions were operated under the following program: 0–2 min, 5% A; 2–28 min, 5–95% A; 28–30 min, 95–5% A. HPLC-MS was operated under the following parameters: electrospray ionization (HESI), positive ion mode (ESI+) and negative ion mode (ESI−) scanning, a voltage of 3.5 kV, a capillary temperature of 320 °C, a probe heater temperature of 350 °C, a sheath gas pressure of 35 arb, and an aux gas pressure of 10 arb.

#### 4.2.3. Animal Experiments

Hepa1-6 cells were purchased from the Chinese Academy of Sciences Cell Bank (Shanghai, China). Cells were cultured in DMEM medium (Servicebio, Wuhan, China) with 10% FBS (Lonsera, Suzhou, China). Five-week-old male C57BL/6j mice were purchased from Jinan Pengyue Experimental Animal Breeding Co., Ltd. (Jinan, China). This study was reviewed and approved by the Experimental Mice Ethics Committee of the Animal Experiment Center of Shandong University of Traditional Chinese Medicine (SDUTCM20220302025). According to the literature research, 30 g of AS and 15 g of CR are clinical dosages [41], so this concentration was set as the moderate dose group of AC. Therefore, the converted dosage for mice was as follows: the tumor-free group (BC group), model group (M group), AS group (6 g/kg), CR group (6 g/kg), and AC group (6 g/kg), with 5 mice per cage. The dosage of FM was determined to be 80 mg/kg as the medium dose based on a review of the literature [9,42]. The converted dosage for mice was as follows: the tumor-free group (BC group), model group (M group), FM-L group (20 mg/kg), FM-M group (40 mg/kg), and FM-H group (80 mg/kg), with 3 mice per cage. Subsequently, 2 × 10^6^ cells in the logarithmic growth phase were prepared into a single-cell suspension in 100 μL of PBS and then inoculated into the right armpit of mice. The tumor volume was measured every 3 days after inoculation. The BC group and M group were given 0.2 mL of distilled water, and the AC group was given 0.2 mL of different concentrations of AC solution by gavage once a day; 5-FU and FM were dissolved in saline solution and sonicated for 3 min to prepare a suspension, and the FM and 5-FU groups were intraperitoneally injected, once every 2 days. A total of 21 days of treatment was given. After therapeutic administration, mice were anesthetized with isoflurane and then euthanized by cervical dislocation.

#### 4.2.4. Hematoxylin and Eosin (HE) Staining and Immunohistochemical (IHC) Experiment

Tumor tissues were fixed with 4% paraformaldehyde, dehydrated, embedded in paraffin, sectioned, and stained using an HE staining kit (Yuanye, Shanghai, China). For the IHC experiment, after antigen retrieval, the sections were blocked and incubated with primary and secondary antibodies, and then the chromogenic agent was added. The staining results were then observed under a microscope (Olympus ckx53, Tokyo, Japan) and photographed.

#### 4.2.5. Cell Viability and Proliferation Assays

HepG2 and LO2 cells were purchased from the Chinese Academy of Sciences Cell Bank (Shanghai, China). Cells were cultured in DMEM medium (Servicebio, Wuhan, China) with 10% FBS (Lonsera, Suzhou, China) at 37 °C with 5% CO_2_ incubator (Thermo, Waltham, MA, USA).

In cell viability assays, HepG2 and LO2 cells (5000 cells/well) were seeded into 96-well plates, and incubated with 0, 10, 20, 30, 40, and 50 μM of FM, Fer-1, and erastin (1.25, 2.5, 5, 10, and 20 μM) for 24, 48, and 72 h. Then they were incubated with 10 μL of 5 mg/mL methyl thiazolyl tetrazolium (MTT; Beyotime, Shanghai, China) at 37 °C for 4 h. After the culture, the medium was removed and 100 μL of DMSO (Servicebio, Wuhan, China) was added. OD 570 nm values were detected using a microplate reader (Thermo Multiskan Sky, Waltham, MA, USA).

A colony formation assay was used to detect cell proliferation, and HepG2 cells (500 cells/well) were seeded into 6-well plates and cultured for 48 h. Then, the medium was discarded, and the cells were cultured with varying concentrations of FM, erastin, and Fer-1 for 2 weeks. When cell colonies could be observed, the cell colonies were fixed and then stained with 1% crystal violet. The count of cell colonies was recorded utilizing ImageJ software (1.53t).

#### 4.2.6. Cell Cycle Assay

HepG2 cells were seeded into 6-well plates and exposed to FM for 48 h. Subsequently, cells were gathered and washed with precooled PBS, and precooled 70% ethanol was added overnight at 4 °C. Then, RNase and PI staining solutions were added and incubated at 37 °C for 0.5 h. The assay was subsequently analyzed by flow cytometry (BD Accuri C6Plus, Franklin Lakes, NJ, USA). The ModFit LT 3.2 software (Verity Software House, New Hampshire, NJ, USA) was used to analyze the percentage of cells at each stage.

#### 4.2.7. Analysis of MMP and ROS 

HepG2 cells were seeded into 6-well plates and exposed to FM for 24 h. MMP and ROS were detected using JC-1 (Solarbio, Beijing, China) and the fluorescent probe DCFH-DA (Beyotime, Shanghai, China). Following a 20 min incubation at 37 °C, the cells were detected by a microscope (Olympus ckx53, Tokyo, Japan) and flow cytometry, with a data analysis conducted using FlowJo-V10 software.

#### 4.2.8. Metabolomics Analysis

Briefly, HepG2 cells were cultured in 10 cm dishes, and after FM treatment, cells were harvested with 2 mL of extraction solvent (methanol: acetonitrile = 3:1, −20 °C). The extraction mixture was moved to 5 mL tubes, sonicated for 10 min (Scientz, Ningbo, China), and then centrifuged for 10 min. After the supernatant was dried in a freeze-dryer, the precipitate was redissolved with 100 μL of extraction solvent. Next, 10 μL of each sample was mixed as a quality control (QC) sample. Chromatographic separation was performed using Q Exactive Orbitrap-MS (Thermo Fisher, Waltham, MA, USA) on a T3 column (Waters, Massachusetts, USA), and mass spectrometer data were obtained. Then, the data were analyzed using the Xcalibur workstation (Thermo Fisher, USA) and Compound Discoverer 3.3 SP2 software (Thermo Fisher, USA) to obtain the quantitative data. Then, a multivariate analysis was performed on the quantitative data using SIMCA-P 14.1 software (Umetrics, Västerbotten County, Sweden) [43]. According to the VIP values (VIP > 1), Fold Change (FC > 1), and *t*-test (*p* < 0.05), differential metabolites were screened out, and MetaboAnalyst 5.0 was used to enrich the metabolic pathway [44].

#### 4.2.9. Analysis of MDA, Fe^2+^, and GSH 

HepG2 cells were seeded in 6-well plates. After the cell adhered, media containing FM, erastin, and Fer-1 were added for treatment for 24 h. The cells were then scraped off and lysed and then centrifuged at 12,000× *g* for 10 min to obtain the supernatant. The MDA Content Assay Kit, GSH Content Assay Kit, and Ferrous Ion Content Assay Kit (Solarbio, Beijing, China) were used to analyze MDA and GSH contents in cells and intracellular iron content according to the instructions.

#### 4.2.10. Western Blotting Assay

HepG2 cells were cultured in 10 cm dishes. After the cells adhere, the medium containing FM was added and cultured for 24 h. Cells were then lysed on ice using RIPA cleavage buffer (Beyotime, Shanghai, China), which was supplemented with protease inhibitors. Then, the loading buffer (Sevenbio, Beijing, China) was mixed and heated in a metal bath. Total protein samples were electrophoresed on SDS-PAGE gels (SparkJade, Jian, China) and then transferred to PVDF membranes (GE, Chicago, IL, USA). After blocking with 5% skimmed milk for 2 h, the membranes were washed with TBST and then underwent incubation with primary antibodies (including Cyclin B1, CDK1, Chk1, Phospho-Chk1, Cdc25C, p53, Histone H2A.X, Phospho-Histone H2A.X (Ser139), GPX4, xCT, and β-actin) at 4 °C overnight. After washing with TBST, the membranes were incubated with secondary antibodies for 1 h at room temperature. After washing with TBST, the protein expressions on the membranes were detected using the ChemiDoc XRS System (Bio-Rad ChemiDoc XRS+, Hercules, CA, USA) and using the ECL detection kit (Millipore, Burlington, MA, USA).

#### 4.2.11. Statistics and Reproducibility

A statistical analysis was performed using GraphPad Prism Software version 9.5 (San Diego, CA, USA). Data were expressed as mean ± standard deviation (SD). One-way ANOVA analysis of variance was used for comparisons among multiple groups, and the *t*-test was used for comparison between two groups. Significance is indicated as follows: * represents *p* < 0.05; ** represents *p* < 0.01; *** represents *p* < 0.001. Data shown are representative of at least three independent experiments, including cell experiments, animal studies, and blots.

## 5. Conclusions

In this study, we confirmed that FM significantly inhibits HCC cells by inducing cell cycle arrest at the G_2_/M phase and causing DNA damage. Additionally, our findings revealed that FM could induce ferroptosis by inhibiting the p53/xCT/GPX4 pathway and regulating glutathione metabolism. In summary, FM has the potential to induce cell cycle arrest and ferroptosis in HCC cells. Nonetheless, more targets and mechanisms remain to be explored, which may provide insights into the mechanism of FM in HCC treatment.

## Figures and Tables

**Table 1:** The interactions between targets and FM.

Target	PDB ID	Resolution	Docking Score	Amino Acid Residue
CHK1	5oq7	2.10 Å	−7.015	ASP-148
Cdc25C	3op3	2.63 Å	−5.317	TRP-414, TYR-485
CDK1	4yc6	2.6 Å	−5.802	ASP-146
CCNB1	5lqf	2.06 Å	−7.888	LEU-83
CCNB2	O95067	-	−5.012	ASN-350, LYS-353
CCNA2	7mkx	3.08 Å	−5.858	LYS-33
KIF11	4a51	2.75 Å	−6.091	TRP-127, ARG-221
AURKB	4af3	2.75 Å	−7.129	LYS-106, ALA-157
FEN1	5fv7	2.84 Å	−4.675	ASP86
BIRC5	2rax	3.3 Å	−3.487	ASP-71

**Table 2:** Differential metabolites associated with FM on HepG2 detected by UPLC-MS/MS.

No.	Metabolites	Formula	*m*/*z*	RT [min]	HMDB	VIP	*p*	FC	Trend	Scan Mode
1	L-Lysine	C_6_H_14_N_2_O_2_	147.1128	1.103	HMDB0000182	1.073	1.70 × 10^−2^	2.36	↑	+
2	Choline	C_5_H_14_NO	105.1075	1.285	HMDB0000097	5.457	1.46 × 10^−2^	3.90	↑	+
3	L-Serine	C_3_H_7_NO_3_	106.0926	1.287	HMDB0000187	1.766	3.60 × 10^−2^	6.13	↑	+
4	Citrulline	C_6_H_13_N_3_O_3_	176.1031	1.295	HMDB0000904	1.629	3.93 × 10^−3^	127.97	↑	+
5	L-Arginine	C_6_H_14_N_4_O_2_	175.1190	1.296	HMDB0000517	1.308	1.12 × 10^−3^	0.06	↓	+
6	L-Threonine	C_4_H_9_NO_3_	120.0658	1.304	HMDB0000167	1.046	1.02 × 10^−2^	0.12	↓	+
7	N-Acetylneuraminic acid	C_11_H_19_NO_9_	308.0993	1.319	HMDB0000230	1.226	1.74 × 10^−2^	0.43	↓	−
8	Asymmetric dimethylarginine	C_8_H_18_N_4_O_2_	203.1504	1.328	HMDB0001539	1.134	5.37 × 10^−3^	18.90	↑	+
9	Uridine diphosphate-N-acetylglucosamine	C_17_H_27_N_3_O_17_P_2_	606.0758	1.639	HMDB0000290	1.183	4.92 × 10^−5^	0.05	↓	−
10	L-Valine	C_5_H_11_NO_2_	118.0865	1.941	HMDB0000883	6.734	9.46 × 10^−4^	2.76	↑	+
11	dGDP	C_10_H_15_N_5_O_10_P_2_	426.0231	1.968	HMDB0000960	2.681	1.33 × 10^−3^	0.03	↓	−
12	L-Methionine	C_5_H_11_NO_2_S	150.0584	2.023	HMDB0000696	5.192	2.34 × 10^−3^	2.51	↑	+
13	L-Isoleucine	C_6_H_13_NO_2_	132.1021	2.081	HMDB0000172	1.996	2.61 × 10^−2^	0.32	↓	+
14	Glutathione	C_10_H_17_N_3_O_6_S	306.0770	2.084	HMDB0062697	2.849	9.29 × 10^−3^	0.07	↓	−
15	Oxoglutaric acid	C_5_H_6_O_5_	145.0981	2.102	HMDB0000208	4.288	1.57 × 10^−3^	0.20	↓	−
16	Hypoxanthine	C_5_H_4_N_4_O	137.0459	2.181	HMDB0000157	8.273	4.04 × 10^−3^	148.68	↑	+
17	5-Oxoproline	C_5_H_7_NO_3_	130.0501	2.254	HMDB0000267	2.655	1.59 × 10^−3^	5.92	↑	+
18	Acetaminophen	C_8_H_9_NO_2_	152.0707	2.287	HMDB0001859	1.962	3.85 × 10^−4^	2.81	↑	+
19	Niacinamide	C_6_H_6_N_2_O	123.0556	2.338	HMDB0001406	4.396	1.61 × 10^−2^	2.61	↑	+
20	Xanthine	C_5_H_4_N_4_O_2_	153.0408	2.433	HMDB0000292	2.371	4.51 × 10^−3^	184.22	↑	+
21	N-Acetyl-L-aspartic acid	C_6_H_9_NO_5_	174.0399	2.524	HMDB0000812	2.366	2.13 × 10^−2^	0.19	↓	−
22	Isocitric acid	C_6_H_8_O_7_	191.0191	2.558	HMDB0000193	1.904	4.84 × 10^−3^	0.13	↓	−
23	L-Tyrosine	C_9_H_11_NO_3_	182.0812	2.590	HMDB0000158	8.759	1.59 × 10^−3^	2.78	↑	+
24	Spermine	C_10_H_26_N_4_	203.2231	2.601	HMDB0001256	1.816	2.70 × 10^−3^	51.58	↑	+
25	Uracil	C_4_H_4_N_2_O_2_	113.0350	2.624	HMDB0000300	1.159	2.42 × 10^−2^	112.62	↑	+
26	L-Glutamate	C_5_H_9_NO_4_	148.0605	2.756	HMDB0000148	1.631	3.03 × 10^−2^	0.05	↓	+
27	L-Glutamine	C_5_H_10_N_2_O_3_	147.0765	2.881	HMDB0000641	1.761	3.05 × 10^−2^	7.46	↑	+
28	L-Cysteine	C_3_H_7_NO_2_S	120.1580	2.909	HMDB0000574	1.099	2.99 × 10^−2^	0.01	↓	−
29	L-Norleucine	C_6_H_13_NO_2_	132.1020	2.931	HMDB0001645	14.419	3.20 × 10^−3^	2.52	↑	+
30	Triethanolamine	C_6_H_15_NO_3_	150.1126	3.208	HMDB0032538	1.102	1.05 × 10^−4^	2.10	↑	+
31	L-Proline	C_5_H_9_NO_2_	116.0710	3.267	HMDB0000162	2.820	2.38 × 10^−2^	2.39	↑	+
32	Spermidine	C_7_H_19_N_3_	146.1652	4.531	HMDB0001257	6.166	3.30 × 10^−4^	4.68	↑	+
33	Oxidized glutathione	C_20_H_32_N_6_O_12_S_2_	611.1457	4.960	HMDB0003337	4.990	1.79 × 10^−3^	0.14	↓	−
34	L-Phenylalanine	C_9_H_11_NO_2_	166.0863	5.044	HMDB0000159	10.928	6.97 × 10^−3^	2.15	↑	+
35	N′-Formylkynurenine	C_11_H_12_N_2_O_4_	237.0871	5.046	HMDB0001200	1.027	6.80 × 10^−5^	6.34	↑	+
36	Adenine	C_5_H_5_N_5_	136.0619	5.128	HMDB0000034	2.159	9.75 × 10^−3^	12.75	↑	+
37	N-Formyl-L-methionine	C_6_H_11_NO_3_S	176.0379	5.258	HMDB0001015	1.437	8.47 × 10^−4^	2.80	↑	−
38	Pantothenic acid	C_9_H_17_NO_5_	218.1032	5.333	HMDB0000210	1.190	3.19 × 10^−4^	2.46	↑	−
39	8-Isoprostaglandin E2	C_20_H_32_O_5_	351.2183	8.836	HMDB0005844	1.806	4.87 × 10^−2^	3.11	↑	−
40	Glycoursodeoxycholic acid	C_26_H_43_NO_5_	448.3076	11.325	HMDB0000708	1.338	1.85 × 10^−2^	0.07	↓	−
41	8,9-DiHETrE	C_20_H_34_O_4_	339.4816	18.790	HMDB0002311	1.830	5.31 × 10^−3^	0.26	↓	+
42	L-Palmitoylcarnitine	C_23_H_46_NO_4_	399.3280	22.009	HMDB0000222	1.753	9.76 × 10^−5^	0.21	↓	−
43	Eicosapentaenoic acid	C_20_H_30_O_2_	301.2179	23.755	HMDB0001999	1.468	3.24 × 10^−2^	14.10	↑	−
44	Arachidonic acid	C_20_H_32_O_2_	303.2335	25.677	HMDB0001043	3.689	1.12 × 10^−2^	12.90	↑	−
45	Palmitic acid	C_16_H_32_O_2_	255.2331	27.686	HMDB0000220	1.442	2.94 × 10^−2^	6.08	↑	−
46	Oleic acid	C_18_H_34_O_2_	281.2491	28.297	HMDB0000207	1.925	3.27 × 10^−3^	4.13	↑	−
47	Stearic acid	C_18_H_36_O_2_	283.2647	30.628	HMDB0000827	1.510	4.62 × 10^−2^	4.64	↑	−

↑ represents increased metabolite levels compared to the control group, ↓ represents decreased metabolite levels compared to the control group, + represents detection in positive ion mode, and − represents detection in negtive ion mode.

## Data Availability

Data will be made available on request.

## Supplementary Materials

The following supporting information can be downloaded at: https://www.mdpi.com/article/10.3390/ijms26062578/s1.

## Abbreviations

HCC	Hepatocellular carcinoma
TCM	traditional Chinese medicine
AC	Astragalus mongholicus Bunge-Curcuma aromatica Salisb.
FM	formononetin
CDKs	cyclin-dependent kinases
CCNB	cyclin B
PPI	protein–protein interaction
MTT	methyl thiazolyl tetrazolium
MDA	malondialdehyde
DEGs	differential expression genes
