# Supplementary material for: Study on the Mechanism of Formononetin Against Hepatocellular Carcinoma: Regulating Metabolic Pathways of Ferroptosis and Cell Cycle
Source: Int J Mol Sci. 2025 Mar 13;26(6):2578. doi: 10.3390/ijms26062578 (PMC11942389; doi:10.3390/ijms26062578)
Supplement: Supplementary file 1 [file ijms-26-02578-s001.zip › ijms-3491444-supplementary.pdf]

Supplementary materials

Study on the Mechanism of Formononetin against Hepatocellular Carcinoma: Regulating Metabolic Pathways of Ferroptosis and Cell Cycle

Ning Bao [1], Zichao Chen [2], Baohong Li [1], Haolin Yang [1], Xiao Li [1,*] and Zhen Zhang [1,*]

[1] Innovation Research Institute of Traditional Chinese Medicine, Shandong University of Traditional Chinese Medicine, Jinan 250355, China; bn990918@163.com (N.B.); libaohong1019@163.com (B.L.); hlin18006399972@163.com (H.Y.)

[2] Experimental Center, Shandong University of Traditional Chinese Medicine, Jinan 250355, China; chenzichao11@126.com

* Correspondence: 60230065@sdutcm.edu.cn (X.L.); 60230006@sdutcm.edu.cn (Z.Z.); Tel.: +86-15668326829 (X.L.); +86-15662712278 (Z.Z.)

Corresponding authors:

Full address: Innovation Research Institute of Traditional Chinese Medicine, Shandong University of Traditional Chinese Medicine, Jinan, 250355, China (X. Li, Z. Zhang)

E-mail: 60230065@sdutcm.edu.cn (X. Li), 60230006@sdutcm.edu.cn (Z. Zhang)

Phone: 86+15668326829 (X. Li), 86+15662712278 (Z. Zhang)

Fax: 0531-89628572 (Z. Zhang)

4. Materials and Methods

4.1.8. Molecular Dynamics Simulation

MMGBSA Binding Free Energy Calculation: The binding free energies between the small molecule and proteins were calculated using the Molecular Mechanics/Generalized Born Surface Area (MM/GBSA) method [1-3]. The specific formula is as follows:

$$\Delta G_{bind} = \Delta G_{complex} - (\Delta G_{receptor} + \Delta G_{ligand})$$
$$= \Delta E_{internal} + \Delta E_{VDW} + \Delta E_{elec} + \Delta G_{GB} + \Delta G_{SA}$$

In Equation (1), $\Delta E_{internal}$, ΔE_{VDW}, and ΔE_{elec} represent the internal energy, van der Waals interactions, and electrostatic interactions, respectively. The internal energy comprises contributions from bond energy (E_{bond}), angle energy (E_{angle}), and torsion energy ($E_{torsion}$). ΔG_{GB} and ΔG_{GA} collectively denote the solvation free energy, where G_{GB} corresponds to the polar solvation free energy and G_{SA} represents the nonpolar solvation free energy. For ΔG_{GB} , the Generalized Born (GB) model developed by Nguyen et al. was employed with the parameter set *igb* = 2 [4]. The nonpolar solvation free energy (ΔG_{SA}) was calculated as the product of the surface tension coefficient (γ) and the change in solvent-accessible surface area (SA):ΔG_{SA}= 0.0072 × ΔSASA [5]. Entropic contributions were omitted in this study due to their high computational cost and limited accuracy, as discussed in reference [1].

Table S1. HCC key genes

Table S2. The active components of *Astragalus mongholicus* Bunge (AS) and *Curcuma aromatica* Salisb. (CR)

Table S3. The targets of *Astragalus mongholicus* Bunge-*Curcuma aromatica* Salisb. (AC) with HCC

Figure S1. Bioinformatics analysis and molecular docking analysis.

Figure S2. UPLC-MS/MS base peak chromatograms of cell samples.

Figure S3. The Volcano plot of differential metabolites

Figure S4. The effect of erastin and fer1 on Hep G2 cells.

Figure S5. The structure of the protein-small molecule complex at the beginning (0 ns) and end (100 ns) of the simulation.

Figure S6. Binding free energy (MM/GBSA) analysis

Table S4. Binding free energy (MM/GBSA) analysis

Table S1. HCC key genes

AURKA	ERCC6L	PRC1	MYBL2	RAD54L	KIF4A	ATAD2	KIF14
RAD51AP1	SKA3	CENPF	GMNN	UBE2T	TOP2A	NCAPH	CDK1
MND1	FANCI	ASF1B	KIF18B	CEP55	HELLS	DLGAP5	BUB1
CENPM	ORC1	KNTC1	FAM64A	KIAA0101	NCAPG	KIF20A	CHEK1
CCNB1	UHRF1	ECT2	ARHGAP11A	KIF23	TK1	RACGAP1	CDCA5

KIF11	CDCA2	RAD51	NDC80	TYMS	MCM4	CDC45	KIF18A
ZWINT	MCM6	CKAP2L	POLQ	GINS2	CDC25C	NEIL3	CENPK
DEPDC1B	DTL	CENPE	FAM83D	TROAP	CENPU	E2F8	ESPL1
ESCO2	PTTG1	PKMYT1	MCM2	CDC7	TACC3	DEPDC1	CDC20
CDKN3	GINS1	UBE2C	BUB1B	HJURP	MAD2L1	CENPI	SGOL1
EXO1	CENPA	FEN1	PLK4	CDT1	SHCBP1	BIRC5	CCNE2
NUF2	OIP5	CDC25A	KIF15	CCNF	SPC25	RRM2	GTSE1
NEK2	ASPM	FOXM1	CDCA7	TTK	CDCA3	PLK1	HMMR
CHAF1A	NUSAP1	MKI67	PARPBP	CCNB2	CDCA8	SKA1	CCNA2
EZH2	CLSPN	MELK	WDHD1	KIF2C	STIL	RFC4	AURKB
ANLN	TRIP13	KIFC1	PBK	DSCC1	CDC6	MCM10	TPX2
E2F7							

Table S2. The active components of *Astragalus mongholicus* Bunge (AS) and *Curcuma aromatica* Salisb. (CR)

ID	Name	OB (%)	DL	PubChem CID
AS-1	Mairin	55.38	0.78	64971
AS-2	Jaranol	50.83	0.29	5318869
AS-3	Hederagenin	36.91	0.75	73299
AS-4	(24S)-24-Propylcholesta-5-ene-3beta-ol	36.23	0.78	15976101
AS-5	Isorhamnetin	49.60	0.31	5281654
AS-6	3,9-Di-O-methylnissolin	53.74	0.48	15689655
AS-7*	Isoferulic Acid	50.83	0.06	736186
AS-8	7-O-methylisomucronulatol	74.69	0.30	15689652
AS-9	Methylnissolin-3-O-glucoside	36.74	0.92	74977390
AS-10	Astrapterocarpan	64.26	0.42	14077830
AS-11	Bifendate	31.10	0.67	108213
AS-12	Formononetin	69.67	0.21	5280378
AS-13*	Astragaloside IV	22.50	0.15	13943297
AS-14	Calycosin	47.75	0.24	5280448
AS-15	Kaempferol	41.88	0.24	5280863
AS-16	Folinic acid	68.96	0.71	135483768
AS-17*	Astragaloside II	46.06	0.13	13996693

ID	Name	OB (%)	DL	PubChem CID
AS-18	Isomucronulatol-7,2'-di-O-glucosiole	49.28	0.62	15689653
AS-19	1,7-Dihydroxy-3,9-dimethoxy pterocarpene	39.05	0.48	5316760
AS-20	(R)-Isomucronulatol	67.67	0.26	10380176
AS-21*	Calycosin 7-O-Glucoside	10.05	0.81	5318267
AS-22*	Ferulic Acid	39.56	0.06	445858
AS-23	quercetin	46.43	0.28	5280343
AS-24*	Astragaloside III	31.83	0.10	441905
CR-1	Hederagenin	36.91	0.75	73299
CR-2	Wenjine	47.93	0.27	101603568
CR-3	Bisdemethoxycurcumin	77.38	0.26	5315472
CR-4*	Neocurdione	36.65	0.08	24836956
CR-5*	Germacron	32.50	0.07	6436348
CR-6*	Curdione	7.00	0.08	6441391
CR-7*	Curcumol	103.55	0.13	14240392
CR-8*	Curcumin	5.15	0.41	969516
CR-9*	β-Pinene	44.77	0.05	15837102
CR-10*	Borneol	81.80	0.05	64685
CR-11*	Lupeol	12.12	0.78	259846
CR-12*	β-Sitosterol	5.84	0.71	222284

*Indicates potential active ingredients supplemented by article

Table S3. The targets of *Astragalus mongholicus* Bunge-*Curcuma aromatica* Salisb. (AC) with HCC

RAD51#	TYMS	CDC45	CDC25C	CDC7#	FEN1#	BIRC5#	CCNE2
CDC25A	NEK2#	TTK	PLK1	CCNB2#	CCNA2#	AURKB	MELK*
AURKA	TOP2A	CDK1	CHEK1	CCNB1#	KIF11#		

#represents AS-independently regulated targets and * represents CR-independently regulated targets

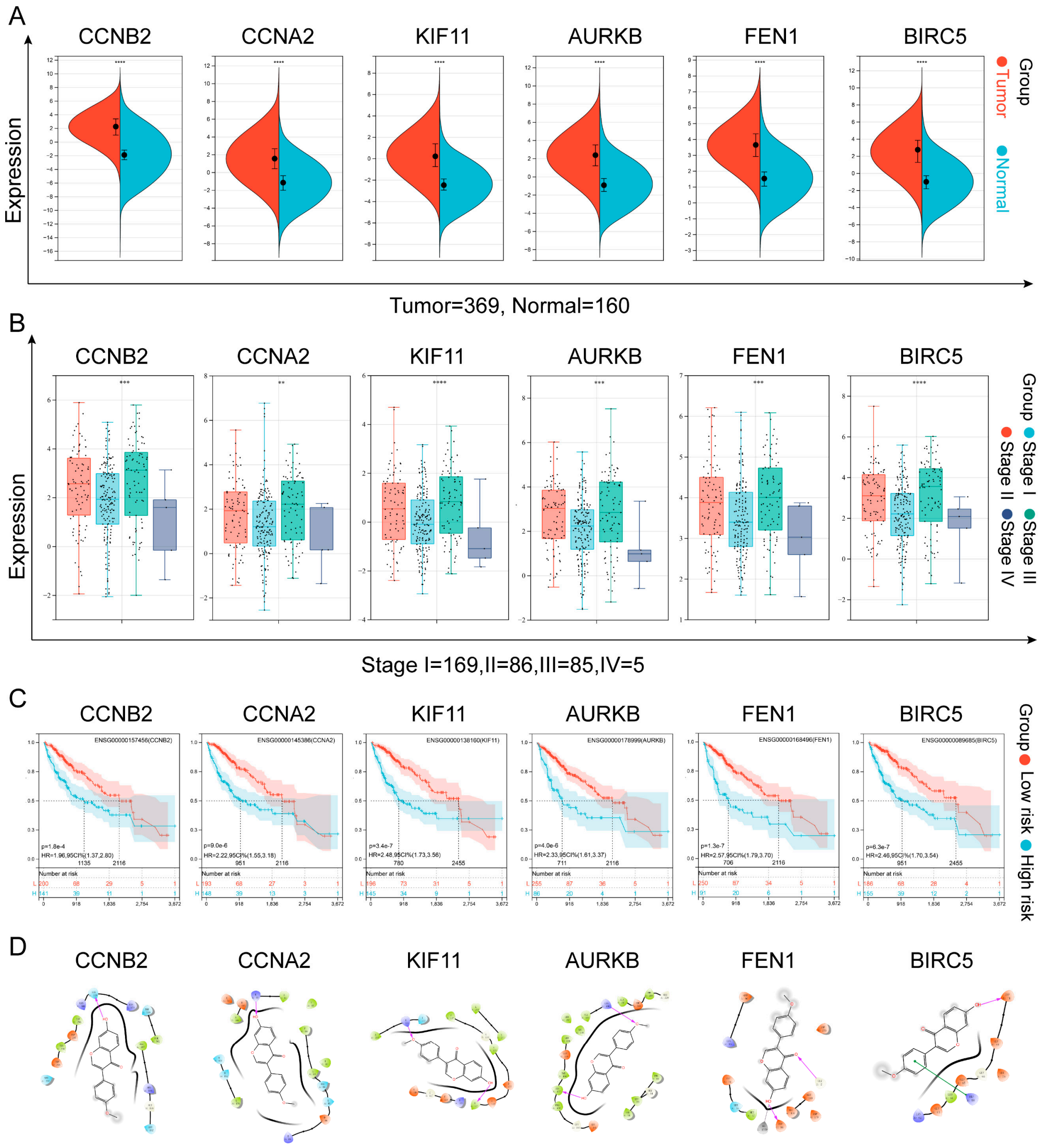

Figure S1. Bioinformatics analysis and molecular docking analysis. (A) Different expression levels of core targets between normal and HCC tissues. Red represents tumor tissue and blue shows normal tissues; (B) Different expression levels of core targets at different stages of HCC; (C) Survival analysis curves for the core targets in HCC patients; (D) 2D interaction diagrams of molecule docking.

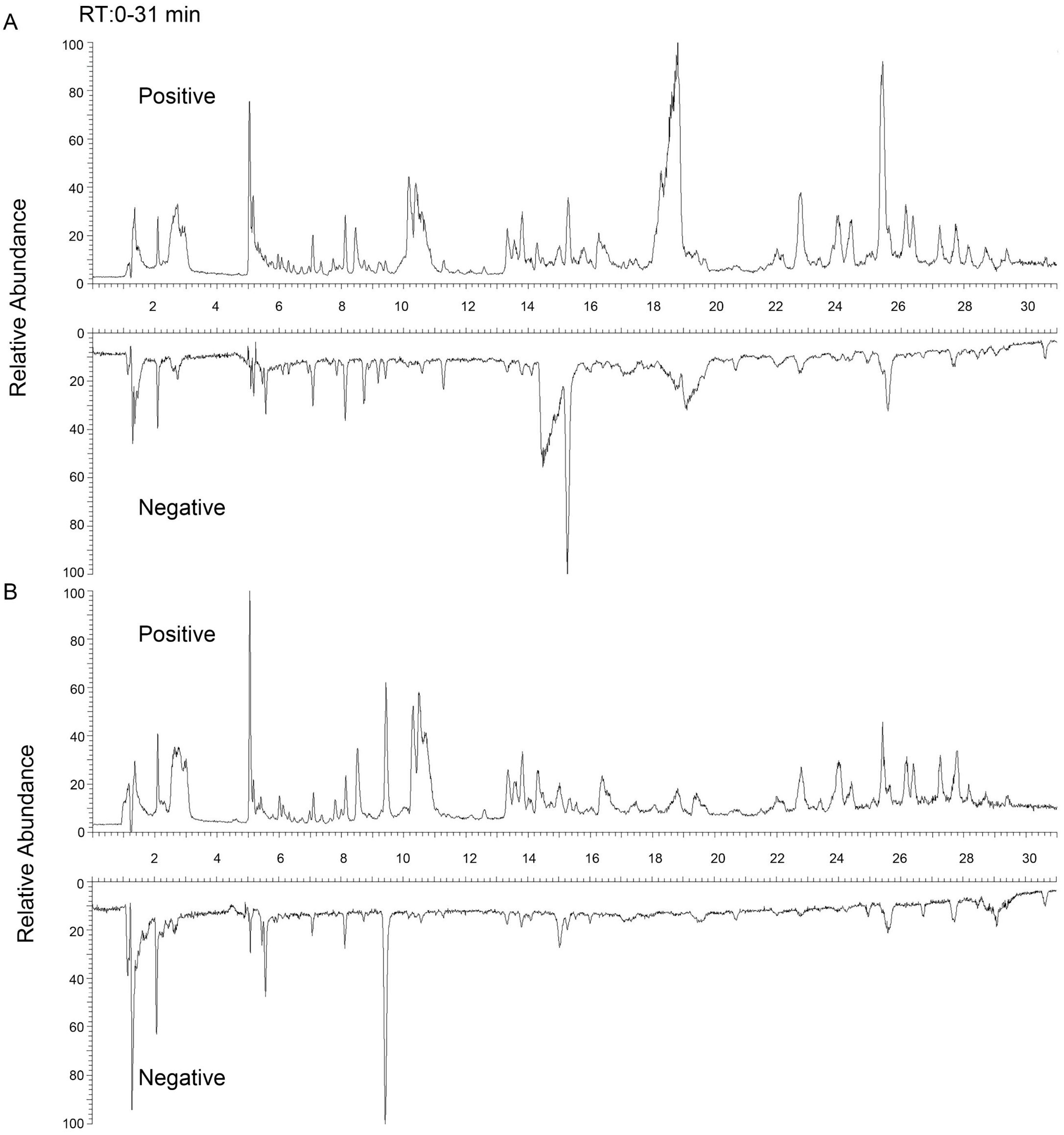

Figure S2. UPLC-MS/MS base peak chromatograms of cell samples. (A) The base peak chromatograms of control group; (B) The base peak chromatograms of FM group.

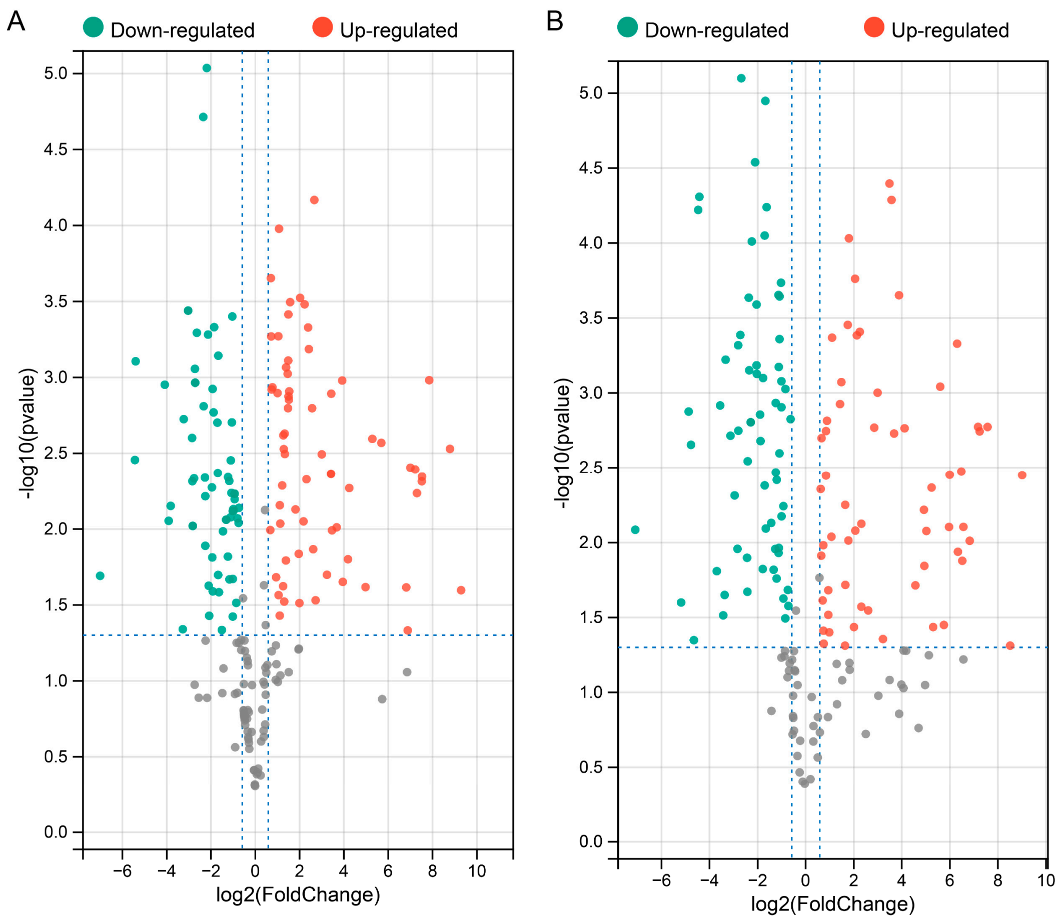

Figure S3. The Volcano plot of differential metabolites

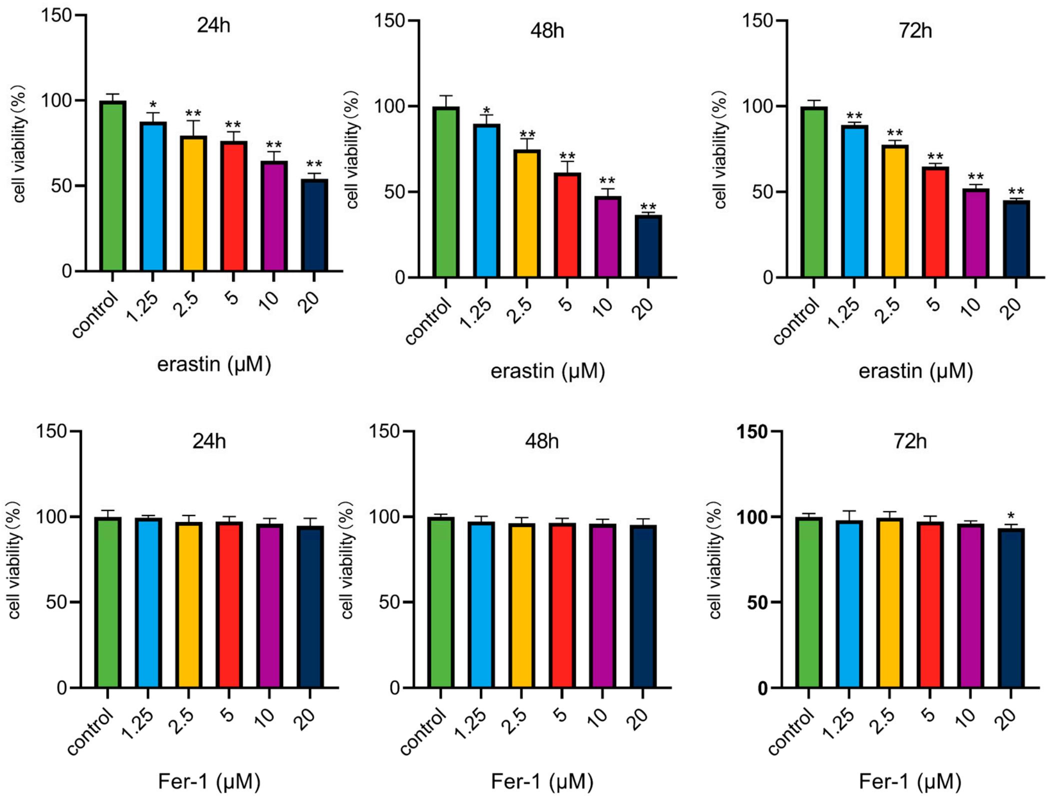

Figure S4. The effect of erastin and fer1 on Hep G2 cells. *n*=5, $^{*}p < 0.05$ and $^{**}p < 0.01$ *vs.* control group.

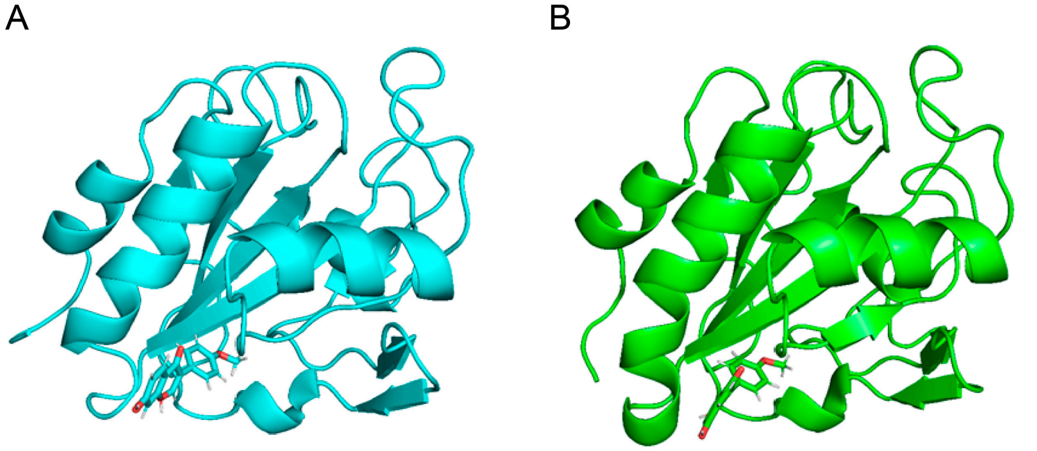

Figure S5. The structure of the protein-small molecule complex at the beginning (0 ns) and end (100 ns) of the simulation. (A) The structure at 0ns; (B) The structure at 100ns.

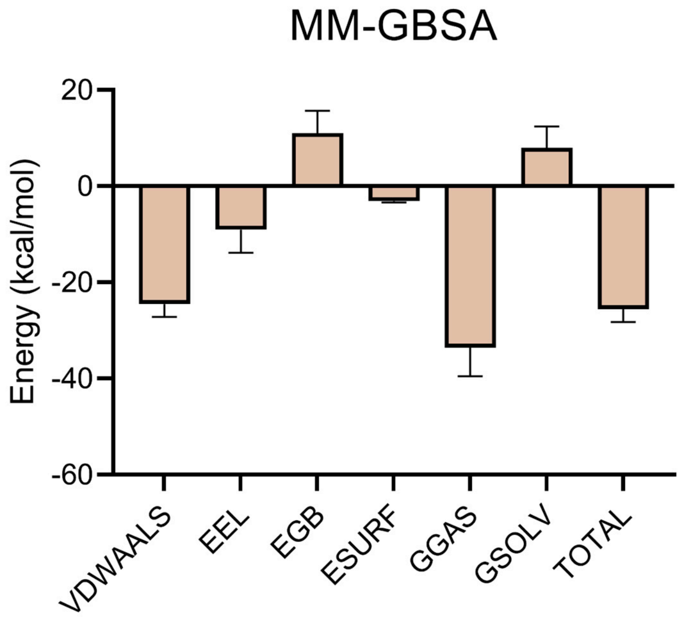

Figure S6. Binding free energy (MM/GBSA) analysis

Table S4. Binding free energy (MM/GBSA) analysis

name	energy（kcal/mol）
$\triangle E_{vdw}$	-24.51
$\triangle E_{ele}$	-9.04
$\triangle E_{GB}$	11.06
$\triangle E_{surf}$	-3.09
$\triangle E_{Gas}$	-33.55
$\triangle E_{solv}$	7.98
$\triangle E_{Bind}$	-25.57

$\triangle E_{vdw}$ is the van der Waals energy, $\triangle E_{ele}$ is the electrostatic energy, $\triangle E_{Gas}$ is the gas phase free energy, $\triangle E_{Gas}=\triangle E_{vdw}+\triangle E_{ele}$; $\triangle E_{surf}$ is the non-polar solvent energy, $\triangle E_{GB}$ is the polar solvent energy, $\triangle E_{solv}$ is the solvent energy, $\triangle E_{solv}=\triangle E_{GB}+\triangle E_{surf}$; $\triangle E_{Bind}$ is the overall binding free energy, $\triangle E_{Bind}=\triangle E_{Gas}+\triangle E_{solv}$.

Reference:

[1] Hou T, Wang J, Li Y, et al. Assessing the performance of the MM/PBSA and MM/GBSA methods. 1. The accuracy of binding free energy calculations based on molecular dynamics simulations [J]. J Chem Inf Model, 2011, 51(1): 69-82. https://doi.org/10.1021/ci100275a

[2] Genheden S, Ryde U. The MM/PBSA and MM/GBSA methods to estimate ligand-binding affinities [J]. Expert

opinion on drug discovery, 2015, 10(5): 449-461. https://doi.org/10.1517/17460441.2015.1032936
[3] Rastelli G, Rio A D, Degliesposti G, et al. Fast and accurate predictions of binding free energies using MM-PBSA and MM-GBSA [J]. J Comput Chem, 2010, 31(4): 797-810.
[4] Nguyen H, Roe D R, Simmerling C. Improved Generalized Born Solvent Model Parameters for Protein Simulations [J]. J Chem Theory Comput, 2013, 9(4): 2020-2034. https://doi.org/10.1021/ct3010485
[5] Weiser J, Shenkin P S, Still W C. Approximate atomic surfaces from linear combinations of pairwise overlaps (LCPO) [J]. J Comput Chem, 1999, 20(2): 217-230.
